# How Electroencephalogram Reference Influences the Movement Readiness Potential?

**DOI:** 10.3389/fnins.2017.00683

**Published:** 2017-12-11

**Authors:** Yuxia Hu, Lipeng Zhang, Mingming Chen, Xiaoyuan Li, Li Shi

**Affiliations:** ^1^Henan Key Laboratory of Brain Science and Brain-Computer Interface Technology, Department of Automation, School of Electric Engineering, Zhengzhou University, Zhengzhou, China; ^2^Department of Automation, Tsinghua University, Beijing, China

**Keywords:** EEG, re-reference, readiness potential (RP), reference electrode standardization technique (REST), common average reference (CAR)

## Abstract

Readiness potential (RP) based on electroencephalograms (EEG) has been studied extensively in recent years, but no studies have investigated the influence of the reference electrode on RP. In order to investigate the reference effect, 10 subjects were recruited and the original vertex reference (Cz) was used to record the raw EEG signal when the subjects performed a motor preparation task. The EEG was then transformed to the common average reference (CAR) and reference electrode standardization technique (REST) reference, and we analyzed the RP waveform and voltage topographies and calculated the classification accuracy of idle and RP EEG segments. Our results showed that the RP waveform and voltage topographies were greatly influenced by the reference, but the classification accuracy was less affected if proper channels were selected as features. Since the Cz channel is near the primary motor cortex, where the source of RP is located, using the REST and CAR references is recommended to get accurate RP waveforms and voltage topographies.

## Introduction

The idea of motor task prediction was first proposed by von Helmholtz (1867), who attempted to explain how humans localize objects (Ahmadian et al., 2013). With the discovery of the brain's electrical activity and the improvement of acquisition equipment performance, predicting motor task became a real possibility. Without a doubt, the interpretation of readiness potential (RP) is meaningful and challenging (Wolpert and Flanagan, 2001; Baker et al., 2012).

The readiness potential is a slow negative potential that can begin as early as 1.5 s before voluntary movement. It features a steeper slope and larger amplitude over the contralateral primary motor cortex (Shibasaki and Hallett, 2006). As a slow cortical potential close to direct-current, the RP is typically not visible in single trial. However, it can be observed clearly using average technology. When the signals are filtered in the 0.1–1 Hz range, the results are better (Garipelli et al., 2011). To date, most of the research on RP has focused on the accuracy of single-trial signal extraction, which can be used in a brain–computer interface. A recent study utilizing a 0.1–1 Hz filter and down-sampling methods to detect self-paced reaching movement intention from EEG signals achieved an average sensitivity of 76 ± 0.07% (Lew et al., 2012). A subsequent study of single-trial RP analysis combing a spatial smoothing filter and common average reference (CAR) reported an average accuracy of 88 ± 0.05% in a contingent cue variation paradigm (Garipelli et al., 2013). In addition, some studies have investigated the use of lateralized RP in combination with imagined movement rhythms to improve the speed and accuracy of brain–computer interfaces (Blankertz et al., 2003, 2006).

Some of the above authors mentioned the effects of the reference; however, none of them discussed how the reference influences RP. Despite the enormous technological advances in the field, an accepted EEG reference is yet still to be settled upon. Ideally, the reference site should be an electrically neutral location, where there are no potential changes; however, there are no truly neutral locations in the human body. In order to reduce the influence of the reference electrode, a number of different reference schemes have been proposed, such as the Cz (Lehmann et al., 1998; Hesse et al., 2004), nose (Andrew and Pfurtscheller, 1996; Essl and Rappelsberger, 1998), linked mastoids or ears (Gevins and Smith, 2000; Croft et al., 2002; Jin et al., 2015), and CAR (Offner, 1950; Nunez et al., 2001). Related studies have indicated that the CAR reference has obtained a large consensus because it is least biased (Srinivasan et al., 1998; Ferree, 2006). Since the surface integral of the electric potential over a volume conductor containing all the current sources is zero, a virtual zero-potential point is provided by the average potential of all the electrodes (Bertrand et al., 1985).

A groundbreaking study of the reference electrode standardization technique (REST) used scalp potentials to determine neural electrical activity and approximately reconstructed the equivalent sources from scalp EEG recordings with a scalp point or average reference, with the potentials referenced at infinity approximately reconstructed from the equivalent sources (Yao, 2001). Although the REST has been shown to be advantageous (Ferree, 2006; Marzetti et al., 2007; Yao et al., 2007; Kayser and Tenke, 2010; Qin et al., 2010; Chella et al., 2016), most recent EEG studies have not used this method, especially for RP, which is sensitive to the reference.

The present study examined the impact of different references (Cz, CAR, and REST) on RP. First, we analyzed the waveform of RP across the three reference electrodes. Next, the reference effect on the activation of brain regions was investigated by drawing the voltage topographies of RP. Finally, the recognition accuracies of the idle and RP states were calculated and compared across the three references.

## Materials and methods

### Participants

Ten healthy subjects (S1–S10, age 26.5 ± 2.1 years, one female, all right-handed) recruited from Zhengzhou University participated in the experiment. All of the participants had normal or corrected-to-normal vision. Prior to the experiment, they were informed of the experimental procedure and signed a letter of consent. The study was approved by the local ethics committee for the Protection of Human Subjects for the Zhengzhou University.

### Experiment paradigm

Each participant was seated in a comfortable chair in a room with normal lighting and temperature. The participant sat facing a screen and was asked to watch the center of the screen. During the recording, the participant was asked to try to avoid eye movement, swallowing, and unnecessary limb movements.

**Figure 1:**
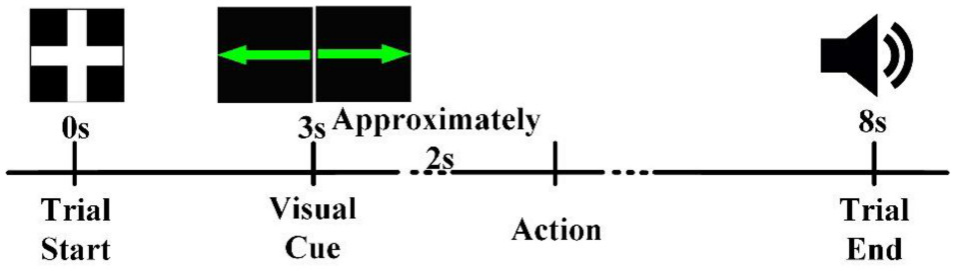
The timeline of a trial. Each trial began in the idle state, in which the participant rested his or her hands, forearms, and elbows on the armrest of a chair and relaxed his or her hands. A visual cue informed the participant that a task should be performed about 2 s later. After completion of the task, an auditory cue instructed the participant to return to the idle state.

At the beginning of each trial, a white cross was presented in the center of the screen (Figure 1). For the next 3 s, participants remained idle, with their hands, forearms, and elbows resting on the armrest of the chair. Next, a green arrow pointing either left or right appeared in the center of the screen for 0.5 s. After the cue disappeared, the participants prepared to perform the corresponding task instructed by the visual cue (left hand movement for left-pointing arrow, right hand movement for right-pointing arrow). After a preparation time of about 2 s, the participants performed the hand movement. Then, five seconds after the visual cue, an auditory cue was presented to inform the participant to return to the idle state. Six sessions were conducted for each participant, with 40 trials per session (20 trials each for left and right).

Two bipolar electrodes were attached to the participant's left and right arm to record electromyograms (EMG) during arm movement.

### EEG recording

**Figure 2:**
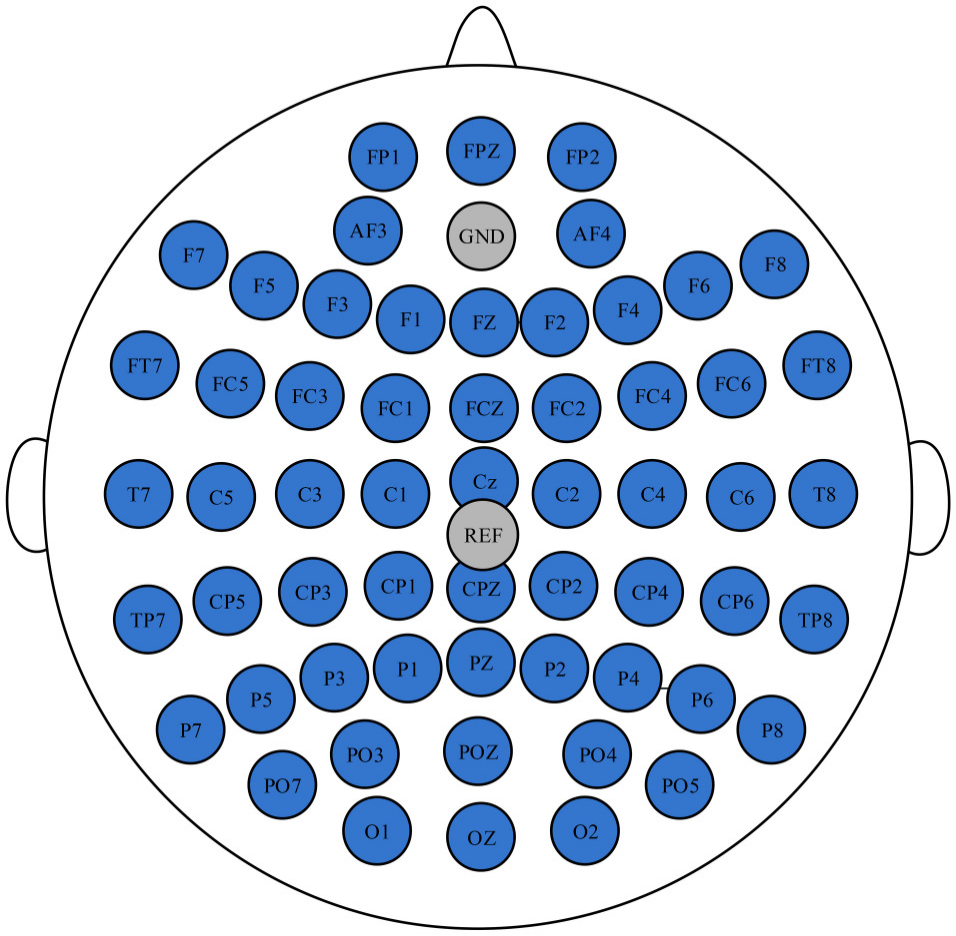
The position of the selected electrodes and the Cz-REF.

EEG data was recorded using a Neuroscan NuAmps digital amplifier system with 58 electrodes arranged in the standard 10–20 EEG configuration. All of the brain regions were covered by these electrodes. Two extended bipolar channels (BP3 and BP4) were used to acquire the left and right arms' EMG signals. The EEG signals were acquired at a sampling rate of 1,000 Hz with the Cz-REF as a reference, and the impedance of all electrodes was less than 5 KΩ. The Cz-REF was located between the Cz and CPZ electrodes. The selected electrodes and the Cz-REF are shown in Figure 2.

### Data preprocessing and re-reference

**Figure 3:**
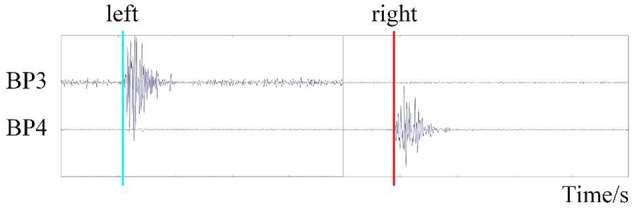
The time of movement onset detection based on EMG.

Data analysis consisted of two parts: EMG analysis and EEG data analysis. We acquired the onset time of hand movement for each trial by processing the EMG data, as shown in Figure 3. The event-related potential, brain voltage topographies, and classification accuracy of the RP state and idle state under different references were acquired by processing the EEG data.

The EMG data were filtered by a basic finite impulse response filter with respective cutoff frequencies of 6 and 50 Hz. We then calculated the energy of the filtered data and set the proper threshold to detect the onset time of motion. We recorded the onset times in a TXT file for EEGLAB toolbox analysis (Delorme and Makeig, 2004).

For the EEG data, EOG artifacts were first removed by Scan 4.5 software (the threshold was set at 60 μV). Then the processed data was exported to EEGLAB for further analysis.

Both the CAR and REST (Yao, 2001, 2017) were conducted offline to generate the re-referenced EEG. The CAR was conducted using the *reref* function from the EEGLAB toolbox (Delorme and Makeig, 2004), and the REST was conducted using the *rest_refer* function (http://www.neuro.uestc.edu.cn/REST/).

### RP analysis

The original and re-referenced EEG were filtered by a band-pass filter (basic FIR filter, 0.1–1 Hz) and segmented into epochs from −3.5 to −3.4 s with respect to the onset time of motor execution (0 s). The data in the interval [−3.5, −3.4 s] was considered the baseline, and baseline correction was conducted for the segmented EEG epoch.

In order to examine the influence of different reference electrodes on the latency, amplitude, and waveform of RP, the RPs were plotted for the three reference methods. Then, we measured the RP's peak amplitude and latency on channels Cz, C1, C2, FC1, FC2, FCz, F1, F2, and Fz under different reference conditions.

### RP topography analysis

Brain voltage topographies can reflect the topological structure of brain activation. It was important for us to analyze the activation areas of the brain and their change over time. However, previous studies usually drew the RP topographies under only one reference method (such as Cz-REF, CAR, or REST) and ignored the influence of different references. In our study, the 0.5 s averaged EEG segments before motion onset were extracted and voltage topographies were drawn every 0.1 s under the three reference methods.

### Feature extraction

**Figure 4:**
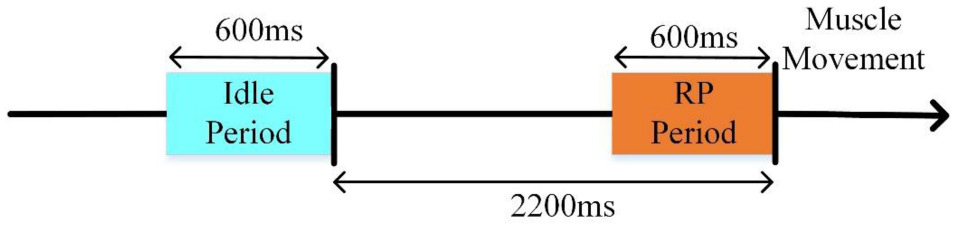
The selected EEG datasets for the RP classifier.

Since RP has obvious time-domain features, we performed classification by the time-domain features. First, the preprocessed data (re-referenced, filtered, segmented, and baseline corrected) were resampled to 10 Hz. Then, the data were divided into two datasets: an idle dataset (i.e., no hand movement) and an RP dataset (i.e., left or right hand movement preparation). We extracted [−2.8 s, −2.2 s] and [−0.6 s, 0 s] of the signals above as idle and RP datasets, respectively (Figure 4).

According to the activated brain regions of RP, the channels (including F3, FZ, F4, FC1, FC2, C3, C4, Cz, CP1, and CP2) were selected for feature extraction in order to ensure the classification accuracy of different subjects. Finally, 60 features were acquired for each trial.

### Classification

The LIBSVM 3.11 toolbox was used for classification (Chih-Chung and Chih-Jen, 2011). The radial basis function kernel was utilized, and the penalty factor (C) and gamma (Γ) parameters were optimized by the grid method. The classification accuracy of the RP and idle state was obtained using 5-fold cross-validation.

## Results

### Reference effects on RP

**Figure 5:**
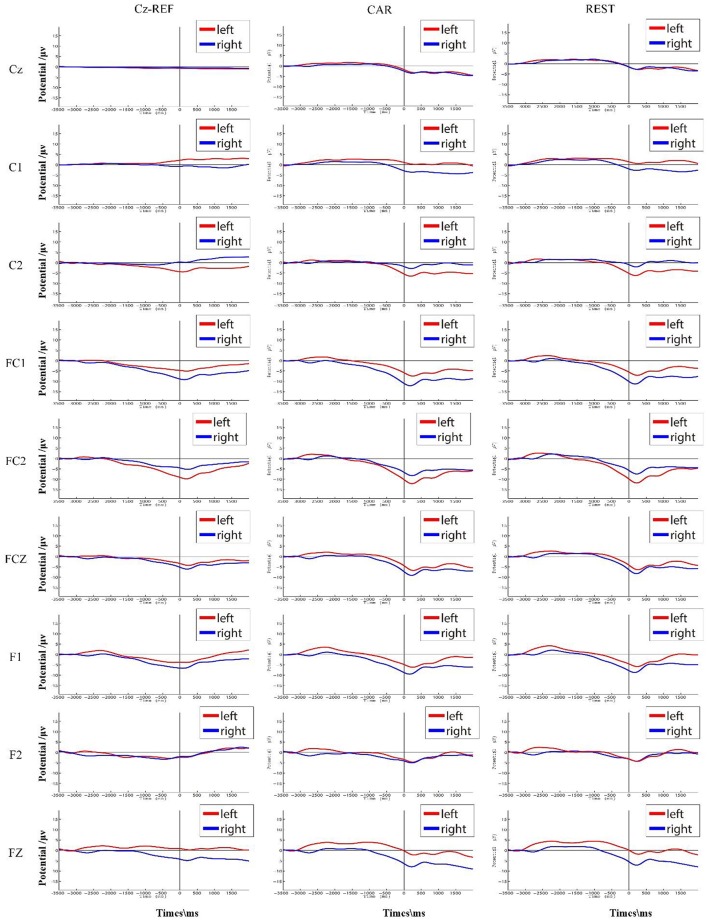
S7's average RP interval (corresponding to [−3.5 s, 2 s] with respect to muscle movement) in three references. The RPs of the left and right hand movements are shown in red and blue, respectively.

Figure 5 shows S7's grand average of the RP interval in three references. When using Cz-REF as a reference, both the left and right RP followed a straight line at the Cz channel, and it was difficult to discern when movement preparation occurred. However, the RP was clear at the Cz channel when the EEG data was adjusted to a CAR or REST reference. For channels C1 and C2, which are near Cz, we can see that the amplitude of RP was smaller from the Cz-REF reference than from the CAR and REST references. The left RP amplitude was higher on channel C2, while the right RP amplitude was higher on channel C1. On channels FC1, FC2, and FCZ, which are located far from Cz-REF, although a clear RP could be seen in all three references, the RP amplitude of Cz-REF was still smaller than those of CAR and REST. On channels F1, F2, and Fz, which are the farthest from Cz-REF, the RP became unclear compared with channels FC1, FC2, and FCZ, and the RP amplitude also became small in the CAR and REST references. The waveforms of CAR and REST were similar for all nine channels, but the amplitude of the REST reference was slightly higher than that of the CAR reference.

### Reference effects on RP voltage topographies

**Figure 6:**
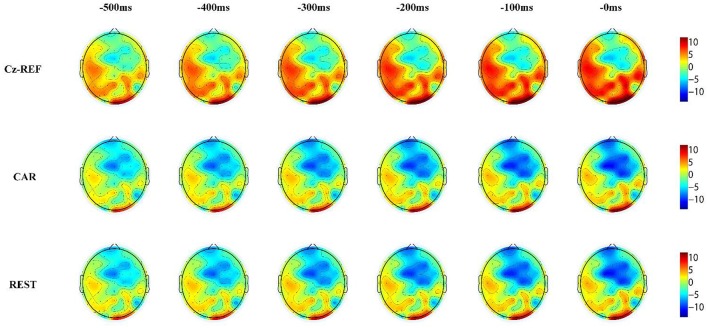
Voltage topographies of S7's left hand movement RP in three references.

**Figure 7:**
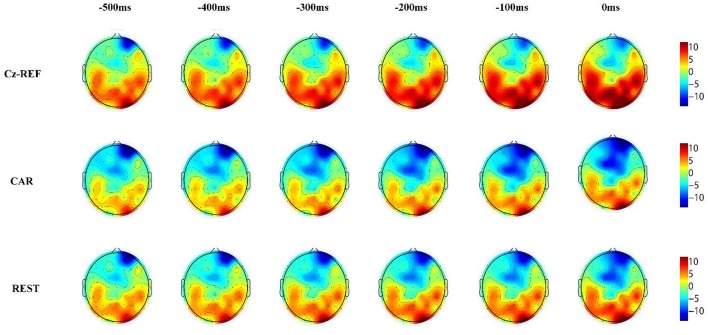
Voltage topographies of S7's right hand movement RP in three references.

Figures 6, 7 show the same subject's RP voltage topographies for the left and right hand movements in the three references. From these two figures, the following three observations can be made. First, the activation areas of the RP were similar. Second, compared with the other two references, the RP voltage topographies of the Cz-REF reference did not show obvious activation, especially for the primary motor cortex. Finally, the REST reference was as good as the CAR reference for drawing the voltage topographies of the RP. The same results were obtained for the other subjects in the experiment.

### Reference effects on classification

Table 1 shows the classification accuracy of the idle and RP periods from 10 participants in three references. High classification accuracy was achieved for all participants except S8, for whom the accuracy was around 70%. The average classification accuracies from the three references were 88.41% (CAR), 87.35% (Cz-REF), and 88.91% (REST), respectively. The REST reference achieved the highest classification accuracy, and the Cz-REF reference achieved the lowest classification accuracy, but the differences among the three references were not statistically significant (*p* < 0.05).

**Table 1:** Accuracy of classification accuracy between idle and RP periods in three references over all 10 participants.

	**S1 (%)**	**S2 (%)**	**S3 (%)**	**S4 (%)**	**S5 (%)**	**S6 (%)**	**S7 (%)**	**S8 (%)**	**S9 (%)**	**S10 (%)**	**Average (%)**
Cz	98.52	83.10	89.66	89.03	86.12	96.01	88.42	70.88	85.23	86.52	87.35
CAR	98.06	87.84	92.67	88.28	91.72	96.08	89.98	67.67	84.00	87.80	88.41
REST	99.07	91.46	91.28	92.83	92.87	96.73	90.01	69.11	82.78	82.78	88.91

## Discussion

The aim of this study was to explore the effects of the reference on RP analysis, including waveforms, voltage topographies, and recognition. As far as we know, this is the first work to compare three commonly used reference schemes—the Cz-REF, CAR, and REST references—in a study of RP. Specifically, we evaluated the amplitude and latency of the RP, then plotted the voltage topographies of the RP and investigated the reference effects on the active area. Finally, the recognition accuracy for the idle and RP periods was calculated over the 10 participants.

We found that the waveform of the RP was influenced by Cz-REF, particularly on the channels near the reference electrode. The main reason for this is that the activation area of motor preparation is close to the Cz-REF reference, and the electrical activity could be conducted to the reference channel. Thus, we could not observe the RP waveform at the Cz channel or even at the C1 and C2 channels near Cz. The RP appeared clearly at the FC1, FC2, and FCz channels due to the longer distance between these channels and the Cz-REF reference. For the F1, F2, and Fz channels, the RP was not better than it was for the FC1, FC2, and FCz channels even though they were the farthest from the reference electrode. We think a plausible explanation is that those channels are not located over the central activation area of the RP (Soon et al., 2008; Andersen and Cui, 2009). There was no significant difference between the CAR and REST references, both of which achieved better results than the Cz-REF reference. Therefore, the CAR and REST references are better choices than the Cz-REF reference when investigating RP.

The RP voltage topographies were also influenced by the reference. With the Cz-REF reference, due to the electrical activity change on the reference electrode, the RP-associated activation brain area could not be observed clearly. However, the activation area could be observed clearly by the CAR and REST references. The RP voltage topographies were almost the same in the CAR and REST references. These results confirmed that the REST reference was as good as the CAR reference for RP voltage topography analysis.

Unlike the RP waveform and voltage topographies, the results of classification accuracy in the three references were similar. The reason may be that although the channels near Cz have disadvantageous features, there are some channels with useful features, such as FC1, FC2, and FCz. High classification accuracy was achieved for all participants except one (S8), for whom the correct rate was around 70%. This could be due to inter-subject differences. Although the REST reference had a slight advantage (0.5% higher than CAR and 1.56% higher than Cz-REF), the difference was not statistically significant. These results indicate that the choice of reference channel does not significantly influence the classification accuracy of the RP and idle EEG segments.

## Conclusion

In conclusion, the results of our research revealed that the RP waveform and voltage topographies were greatly influenced by the reference, but the classification accuracy was less affected if proper channels were selected as features. Since the Cz-REF reference is near the primary motor cortex, where the source of RP is located, the REST and CAR references are better choices for obtaining accurate RP waveforms and voltage topographies.

## Author contributions

YH, LZ, MC, XL, and LS conceived and designed the experiments. YH, LZ, and XL performed the experiments and analyzed the data. YH and LZ wrote the main manuscript text. All authors reviewed the manuscript.

### Conflict of interest statement

The authors declare that the research was conducted in the absence of any commercial or financial relationships that could be construed as a potential conflict of interest.
